# Evaluation of the scolicidal activities of eugenol essential oil and its nanoemulsion against protoscoleces of hydatid cysts

**DOI:** 10.1371/journal.pone.0259290

**Published:** 2021-11-11

**Authors:** Maria Naged Maurice, Enas Abdelhameed Mahmoud Huseein, Mohamed El-Salahy M. M. Monib, Fahd M. Alsharif, Nader Ibrahim Namazi, Alzahraa Abdelraouf Ahmad

**Affiliations:** 1 Faculty of Medicine, Department of Medical Parasitology, Assiut University, Assiut, Egypt; 2 Faculty of Pharmacy, Department of Pharmaceutics and Ind. Pharmacy, Al-Azhar University, Assiut, Egypt; 3 Department of Pharmaceutics and Pharmaceutical Technology, College of Pharmacy, Taibah University, Madinah, Saudi Arabia; Beni Suef University Faculty of Veterinary Medicine, EGYPT

## Abstract

**Background:**

Cystic echinococcosis caused by the cestode *Echinococcus granulosus* remains a serious helminthic zoonosis affecting humans and animals in many endemic developing countries. Surgical intervention is the best management choice, although it is associated with high recurrence rates and serious complications. Also, the commonly used chemotherapeutics exhibited serious side effects. This study aimed to evaluate the protoscolicidal effects of eugenol (Eug) essential oil and its nanoemulsion (Eug-NE) against protoscoleces (PCs) of hydatid cysts *in vitro*.

**Methods:**

Eug-NE was prepared and characterized. Their cytotoxicity on macrophages was assessed by the 3-(4,5-dimethylthiazol-2-yl)-2,5-diphenyltetrazolium bromide assay. *E*. *granulosus* PCs were treated with various concentrations of Eug and Eug-NE at different exposure times. The viability of protoscoleces was evaluated by the eosin exclusion test, and the changes in the morphology of protoscoleces were assessed. Albendazole (ABZ) was used as a positive control.

**Results:**

The cellular cytotoxicity of Eug and Eug-NE on macrophage cells, in minimum and maximum concentrations (0.2 and 1 μl/mL), were nearly negligible ranging from 4.7% to 8.3% and 3.7% to 7.2%, respectively. The results showed highly significant activity of Eug-NE and Eug against hydatid PCs compared to ABZ (*P* < 0.05). Eug and Eug-NE have similar protoscolicidal effects at all used concentrations. Their highest scolicidal activity (100% mortality rate) was recorded at 1 μl/ml after 30 min incubation (LC_50_ = 0.298—LC_90_ = 0.521 and LC_50_ = 0.309—LC_90_ = 0.646, respectively). Both formulations showed time- and dose-dependent effects.

**Conclusions:**

This study suggested the potent scolicidal activities of Eug and Eug-NE as promising alternative scolicidal agents. Future studies are recommended to explore the mechanism of action and treatment response *in vivo* and clinical settings.

## 1. Introduction

Cystic echinococcosis (CE) is one of the most important helminthic zoonosis in humans and animals caused by the larval stage of *Echinococcus granulosus* [1,2]. It is distributed worldwide with high endemicity in developing countries, the Middle East, and North Africa, including Egypt [3]. CE is characterized by the long-term development of the larval stage (hydatid cyst) of *E*. *granulosus* in different organs of the intermediate host, affecting mainly the liver and lungs [4,5]. It is considered a potentially lethal health hazard in 4.6% of the patients if left untreated [6]. It affects more than 1 million people worldwide, with an economic burden exceeding $3 billion per year in management besides the annual livestock losses [7].

Surgery is the best option for CE management. However, laceration, spillage of cyst content, risk of anaphylaxis, and increased risk for recurrence are the main complications that necessitate developing alternative strategies or chemotherapeutics, particularly for surgery-intolerant patients [8]. Benzimidazole derivatives, such as albendazole (ABZ) and mebendazole, are widely used in CE treatment as an alternative or adjuvant to PAIR technique or surgery according to the site and stage of cysts [9]. However, the reported efficacy of ABZ is ~50%, with an overall cure rate not exceeding 30% to 48% [10]. Many patients have demonstrated unsatisfactory results with ABZ chemotherapy [11,12]. Serious side effects have also been reported with chemotherapy, such as liver toxicity, bone marrow suppression, encephalitis syndrome, influenza-like syndrome, and many other hematological disorders [13,14]. Moreover, with the long course of treatment, ABZ could develop a protoscolicidal resistance evidenced by the recurrence commonly observed after treatment cessation [15]. Hence, developing alternative, safe scolicidal agents with higher efficacy rates from medicinal plants and traditional essential oils is highly encouraged by many researchers especially in areas with high endemicity as in North Africa [6,16,17]

Eugenol (Eug) is a naturally occurring aromatic phenol essential oil extracted from *Syzygium aromaticum* (cloves), *Cinnamomum verum* (cinnamon), *Laurus nobilis* (bay leaf), *Myristica fragrans* (nutmeg), and *Ocimum basilicum* (basil) [18]. Eug shows better effectiveness when administered orally due to its rapid intestinal absorption [19]. Eug oil is described as a promising chemotherapeutic agent with different activities, including anti-inflammatory, antioxidant, and different antimicrobial effects [20–22]. However, its antiparasitic activity is less investigated compared to its multifunctionality. Anthelmintic activity of Eug against adult worms of *Fasciola gigantica*, *Schistosoma mansoni*, *Gyrodactylus sp*., and *Haemonchus contortus* has been previously documented [23–26]. It also shows antiprotozoal activity against *Giardia lamblia*, *Plasmodium falciparum*, *Trypanosoma cruzi*, *Leishmania amazonensis*, *Leishmania major*, and *Leishmania donovani* [27–32] besides its insecticidal and acaricidal effects [33,34]. In addition, recent studies reported synergic effects of Eug or its analogs when used in hybridization with other standard drugs used in the management of visceral leishmaniasis and trypanosomiasis [35,36].

Indeed, novel drug formulations and the recent advent of nano preparations have increased drug efficacy in disease control. Nanoemulsions are recently introduced as suitable carriers of active essential oils due to their easy preparation, simple structure, higher thermodynamic stability, and cost-effectiveness accessible for production on an industrial scale [37]. Eug nanoemulsion (Eug-NE) was investigated previously as an antimicrobial agent against *Listeria monocytogenes* and *Salmonella enteritidis* [38] and as an antifungal agent against *Fusarium oxysporum* [39]. Besides its recent application as a larvicide against *Aedes aegypti* larval mosquitoes [40]

This study was designed to investigate the possible scolicidal activities of Eug essential oil and Eug-NE on *E*. *granulosus* protoscoleces (PCs) compared to ABZ *in vitro*.

## 2. Materials and methods

### 2.1. Chemicals

Eug (4-allyl-2-methoxyphenol; commercial-grade; >98%, MW 164.20), 3-(4,5-dimethylthiazol-2-yl)-2,5-diphenyltetrazolium bromide (MTT) reagent, and RPMI 1640 medium were purchased from Sigma-Aldrich (Germany). ABZ was donated by EPICO (Egypt). The biochemical reagents [Tween 20, dimethyl sulfoxide (DMSO), and phosphate-buffered saline (PBS)] were purchased from Alpha Chemical (Cairo, Egypt). Murine macrophage cells Raw 264.7 were provided by VACSERA, Cell Culture Unit, Egypt.

### 2.2. Preparation of ABZ and Eug

DMSO was used as a solvent with a final concentration of 0.1% with no scolicidal effects. ABZ and Eug essential oil (>98%, MW 164.20) were dissolved in 0.1% DMSO, and the stock solution was prepared to reach a final concentration of 800 μg/ml for ABZ [41] and 100 μl/ml for Eug [27,42]. The mixture was adequately homogenized by a magnetic stirrer. Serial dilutions were subsequently done to obtain Eug at 0.2, 0.4, 0.6, 0.8, and 1 μl/ml.

### 2.3. Preparation of Eug-NE 10%

Eug-NE 10% was prepared using the high-pressure homogenization method, as described previously [43]. In brief, Eug oil-in-water emulsion was prepared at room temperature. Tween 20 used as a surfactant was dissolved in distilled water at 5% (v/v). Using an ultrasonic homogenizer (Bandelin SONOPULS HD 2200, Germany) at 20,000 rpm, Eug oil was added dropwise to the aqueous solution of Tween 20 to reach 10% concentration. The process continued for 10 min to confirm stability. The formed Eug-NE (10%) was stored in a well-closed container under refrigeration.

### 2.4. Characterization of Eug-NE

#### 2.4.1. Measurement of nanoemulsion droplet size, polydispersity index (PDI), and zeta potential

The mean droplet size of Eug-NE was measured using the dynamic light scattering method using a Zeta-sizer (Malvern Instruments, UK) at room temperature. The samples were placed in disposable polystyrene cuvettes, and the scattering intensity was measured. All experiments were conducted in triplicates, and the average data were presented as mean ± standard deviation (SD).

#### 2.4.2. Transmission electron microscopy (TEM)

Microscopic observation of Eug-NE was performed using TEM. In brief, diluted Eug-NE (20 μl) was placed on a film-coated 200-mesh copper specimen grid for 10 min, and the excess fluid was removed. The grid was stained with 3% phosphotungstic acid and left to dry for 4 min [39]. The dried coated grid was examined under a TEM microscope (JEOL-JSM-5400 LV) at the Electron Microscope Unit of Assiut University.

#### 2.4.3. Fourier transform infrared (FTIR) spectroscopy

To investigate the possible molecular interactions of Eug-NE, FTIR was performed. In FTIR, the freeze-dried samples at −30° C were used. Particles of the frozen dispersion were lyophilized for 48 h in an Alpha 1–4 LSC Plus freeze dryer (Martin Christ, Germany) operating at a 0.1 mbar vacuum level and a temperature of −52° C [44]. Intermolecular interactions between all ingredients were monitored using a Nicolet iS10 FTIR spectrometer (Thermo Fisher Scientific, Inc., MA, USA). Data were analyzed using OMNIC [38]. The Eug-NE sample was prepared by adding potassium bromide, mixed, and compressed under hydraulic pressure. Infrared spectra were recorded at the wavenumber of 500 to 4000 cm^−1^, with a resolution of 4 cm^−1^.

### 2.5. Cellular cytotoxicity of Eug and Eug-NE 10% (MTT assay)

The MTT assay was performed to determine the cellular toxicity of various Eug and Eug-NE 10% concentrations used in this study. Macrophage cells Raw- 264.7 were cultured in RPMI 1640 medium with FBS 10%, 100 U/ml penicillin, and 100 mg/ml streptomycin and incubated in a 5% CO_2_ atmosphere at 37°C. Macrophages were treated with Eug and Eug-NE at 0.2, 0.4, 0.6, 0.8, and 1 μl/ml. The cytotoxic assay was performed according to Kar Mahapatra et al. [45]. The optical observation of the samples was measured using an enzyme-linked immunosorbent assay reader at a wavelength of 570 nm. The percentage of cell viability was measured as follows [46,47]:

Survivalrate=(AT‐AB)/(AC‐AB)×100,

where AB = blank well optical absorption, AC = control well optical absorption, and AT = treated cells.

### 2.6. Preparation of parasite material and PC collection

Hydatid cysts were obtained from the livers and lungs of infected slaughtered camels at local slaughter houses in Assiut, Egypt. The cysts were transferred to the Parasitology Laboratory, Faculty of Medicine, Assiut University. PCs were collected under aseptic precautions, as described previously [6]. Cyst fluid was aspirated by aseptic puncture and left to precipitate in 45 ml Falcon tubes for 1 h at room temperature without centrifugation, and the supernatant was removed. PC viability was evaluated by the eosin exclusion test. About 10 μl of 0.1% aqueous eosin stain was mixed with 10 μl of the protoscoleces’ pellet and examined under a low-power microscope after 5 min. Dead PCs absorbed eosin and were colored red, but alive PCs remained unstained. All samples with >95% viability were included in the experiment [14].

### 2.7. Determination of scolicidal activities of Eug and Eug-NE 10%

Viable PCs (3000/ml) were cultivated in PBS (pH 7.4) without the addition of either antibiotics or antifungals at 37°C, as described previously [48]. The tested PCs were grouped as follows: (i) Eug at final concentrations of 0.2, 0.4, 0.6, 0.8, and 1 μl/ml; (ii) Eug-NE 10% at final concentrations of 0.2, 0.4, 0.6, 0.8, and 1 μl/ml; and (iii) ABZ sulfoxide as a positive control at 800 μg/ml. Negative control PCs were incubated in PBS with 1 μl/ml DMSO. The experiment was performed in triplicate, in which the content of the tubes was mixed slowly, incubated at 37°C, and checked for viability after 30 min, 1 h, 2 h, and then 2 h intervals until all PCs became dead. After incubation, viability was assessed by the eosin exclusion test, and the protoscolicidal fatality rate (PFR) was determined using the following formula [46]:

$$\mathrm{PFR}\;(\%)=\frac{\mathrm{number}\;\mathrm{of}\;\mathrm{dead}\;\mathrm{PCs}}{\mathrm{number}\;\mathrm{of}\;\mathrm{living}\;\mathrm{PCs}\;(\mathrm{control})}\times 100$$

### 2.8. Structural and ultrastructural studies

At the end of the experiment, an ultrastructural study using a scanning electron microscope (SEM) was conducted. The treated PCs were fixed in 2.5% glutaraldehyde (in 100 mM sodium cacodylate buffer; pH 7.2) for 2 h at room temperature, fixed in 100 mM sodium cacodylate buffer (pH 7.2) for 2 h at room temperature, and processed for SEM. Later, the specimens were washed in distilled water and treated with 1% uranyl acetate for 30 min. Further, the samples were washed thoroughly with PBS and sequentially dehydrated by incubation in ascending ethanol concentrations. The dehydrated samples were immersed in hexamethyldisilazane, air-dried under a fume hood, sputter-coated with gold, and examined by a JEOL 840 SEM operating system at 25 kV [49].

### 2.9. Statistical analysis

Data were verified, coded, and analyzed using IBM-SPSS 24.0 (IBM-SPSS, Inc., Chicago, IL, USA). The results were expressed as the mean ± SD and percentages. One-way analysis of variance (ANOVA) and repeated-measures ANOVA were applied to evaluate the mean differences between the studied groups. Spearman’s rank correlation analysis was used to test the association between variables. LC values were calculated using probit analysis. *P* < 0.05 was considered significant.

## 3. Results

### 3.1. Characterization of Eug-NE

#### 3.1.1. Eug-NE droplet size and PDI

**Fig 1:**
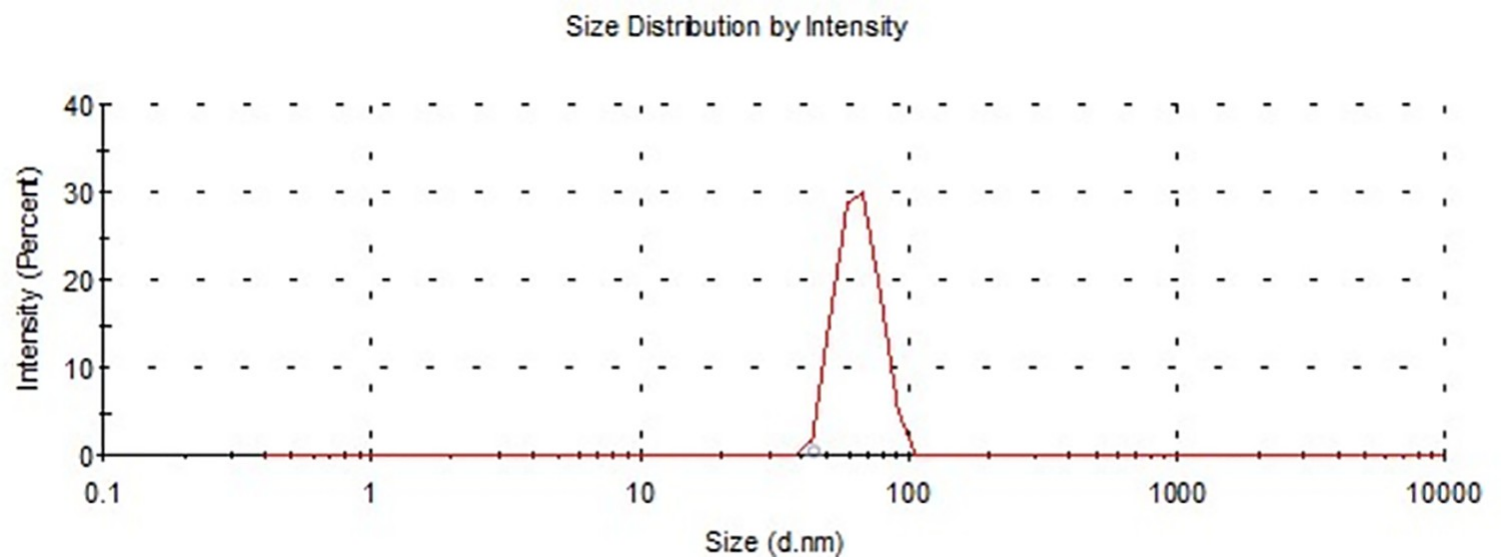
Size distribution of Eug-NE using a Zeta-sizer.

Eug-NE dispersion was a homogeneous system of light milky color when observed macroscopically. The particle size measurements showed that the average particle size and PDI values of Eug-NE were 65 ± 11.11 and 1.00 nm, respectively. However, Eug-NE showed a smaller particle size, and the PDI was high, as shown in Fig 1. This suggested the poor homogeneity of the prepared Eug-NE that may be attributed to some nanodroplets in aggregates.

#### 3.1.2. TEM

**Fig 2:**
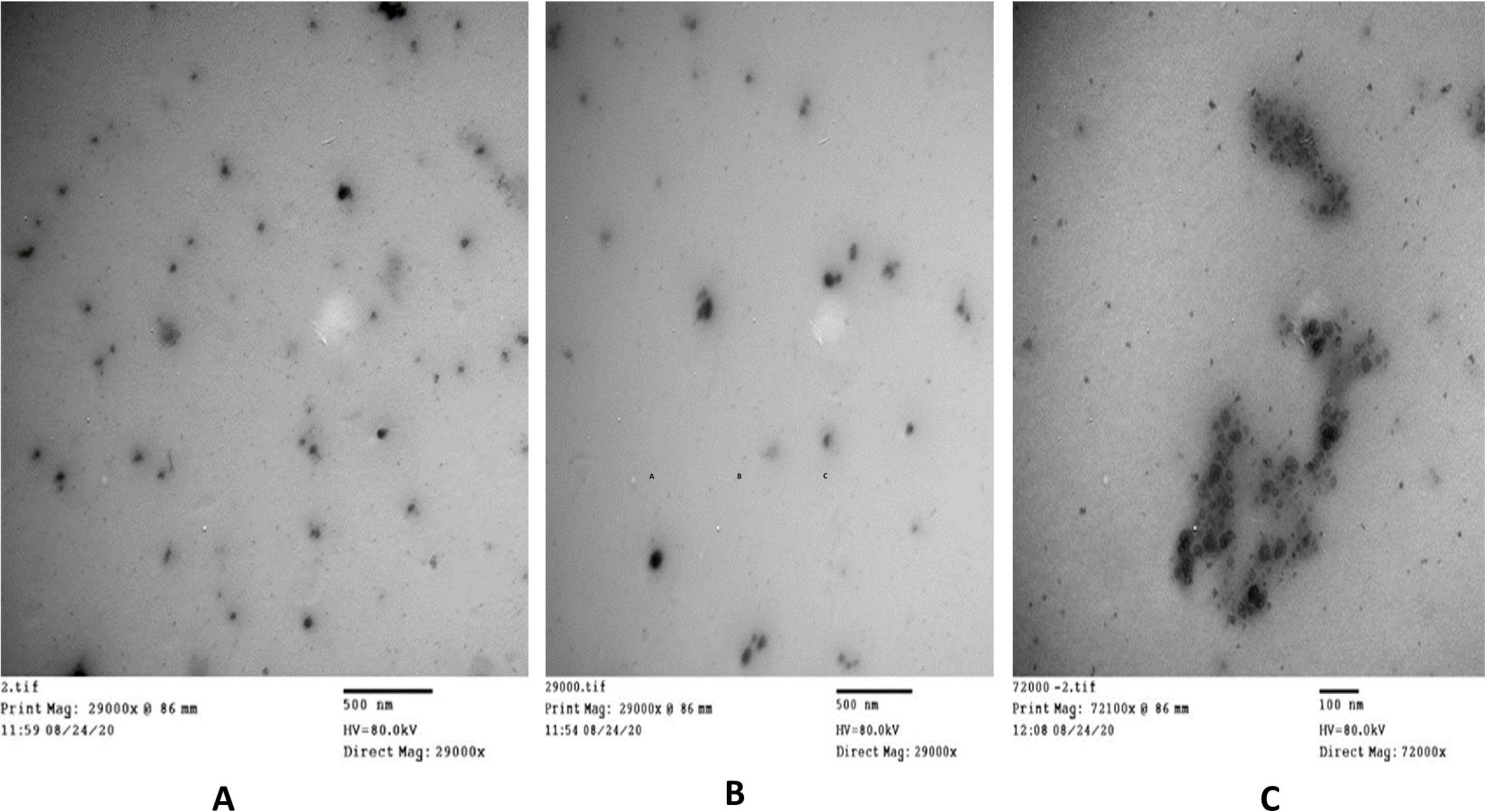
TEM analysis of the formulated Eug-NE.

The size and morphology of the prepared Eug-NE droplets were observed using TEM. TEM images revealed that Eug-NE nanodroplets were nearly spherical in shape, variable in size, and a diameter ranging from 20 to 80 nm. The size measured by TEM agreed with the average droplet size determined by a Zeta-sizer, as elucidated previously (~65 nm; Fig 2A and 2B). However, some Eug-NE droplets existed in aggregates (Fig 2C). This may explain the polydispersity of Eug-NE droplets as indicated by the higher PDI value detected by the light scattering technique (photon correlation spectroscopy). The results suggested that higher PDI values might reflect less stability of the formulation [50]. For this reason, a cosurfactant, such as lecithin, may enhance the stability of the formulation.

#### 3.1.3. FTIR spectroscopy

**Fig 3:**
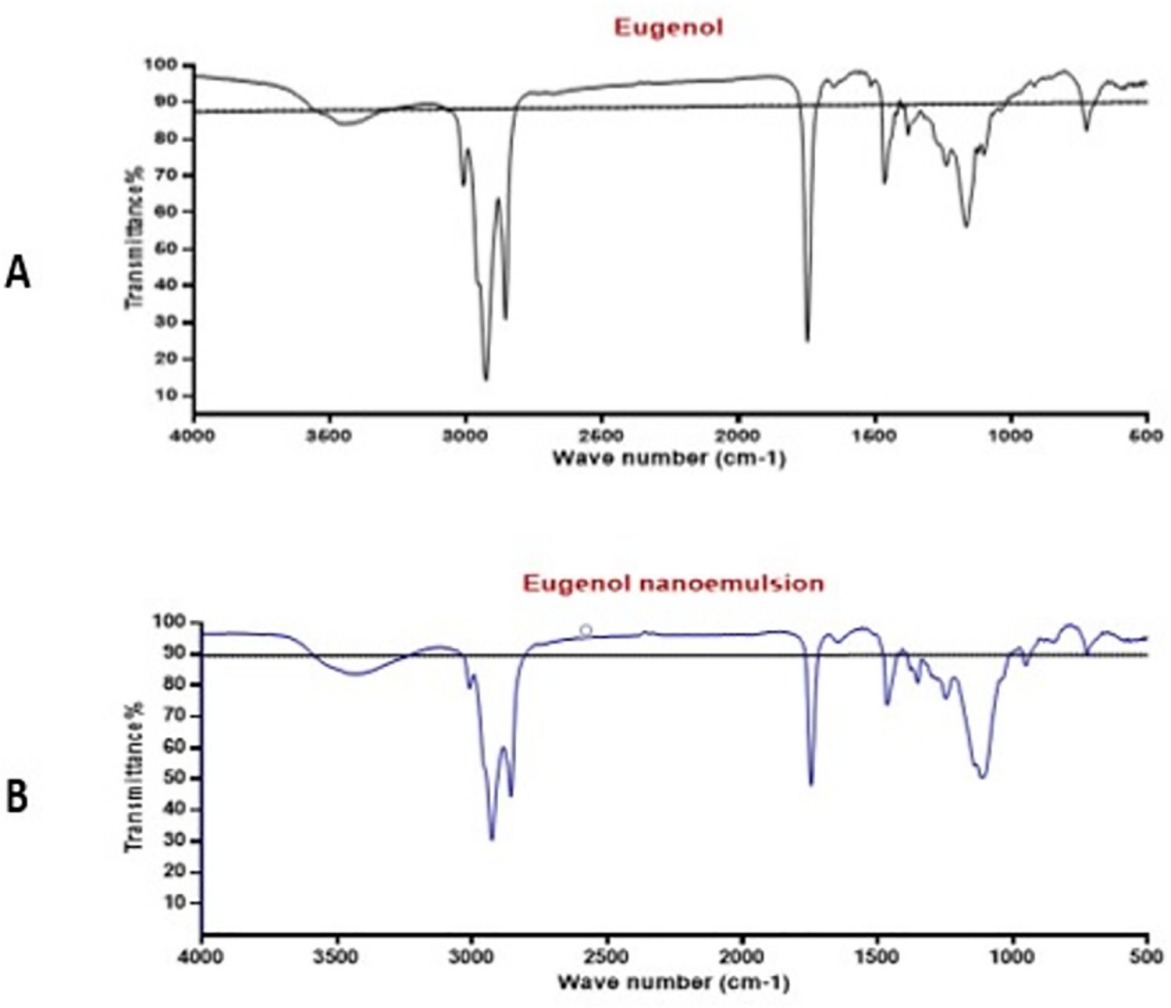
FTIR spectra of Eug and formulated Eug-NE.

FTIR spectra of Eug and Eug-NE were recorded and compared for any possible chemical interaction, as shown in Fig 3A and 3B. A strong band that appears at ~3440 cm^−1^ in both FTIR spectra of Eug and Eug-NE can be argued to be the stretching vibrations of the phenolic O-H group in Eug. Besides, the characteristic absorbance peak found at ~3008 cm^−1^ was correspondent to the C-H stretching peak of the allyl group (C-H attached to olefin) in Eug. Moreover, sharp peaks at ~1700, 1610, and 1465 cm^−1^ were also found with Eug, which can be due to the C = C stretching of the aromatic moiety. These data were consistent with previous reports [51,52]. Similarly, the same peaks were also observed in the Eug-NE spectrum (Fig 3B), indicating the absence of a chemical interaction between Eug and Tween 20 in the nanoemulsion formulation. Further, no new peaks were found, suggesting the absence of interaction.

### 3.2. Cellular cytotoxicity of Eug and Eug-NE (MTT assay)

The Eug cytotoxicity levels were 4.7% to 8.3%, with cell viability of 95.3% to 91.67% at 0.2 and 1 μl/ml, respectively. The Eug-NE cytotoxicity levels were 3.7% to 7.2%, with cellular viability of 96.30% to 92.60% for the same concentrations. The cytotoxic effect of Eug-NE was lower than Eug without statistical significance (*P* > 0.05). Therefore, the toxicity level of Eug and Eug-NE was negligible.

### 3.3. PC viability

**Fig 4:**
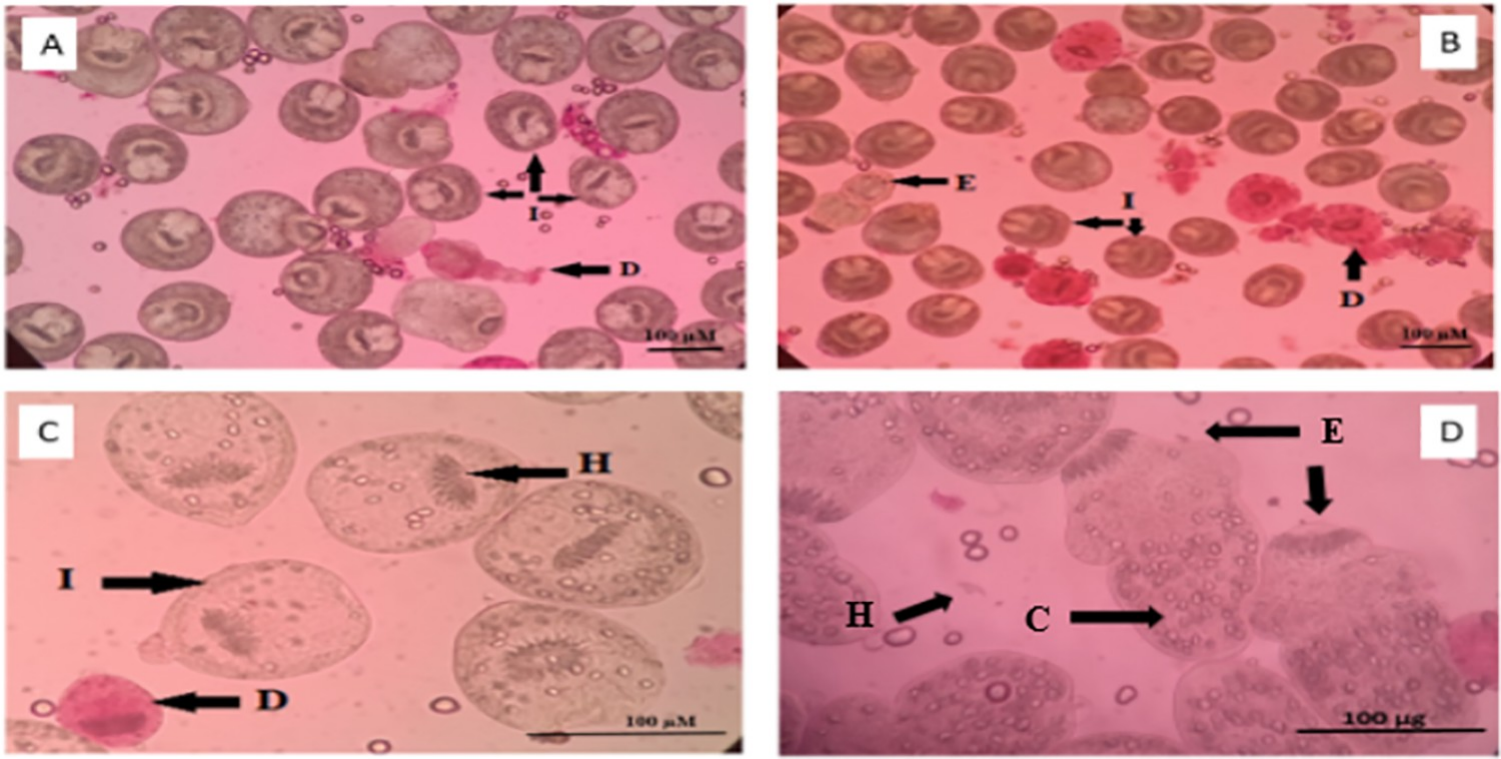
Assessment of PC viability by the eosin exclusion test. Light microscopy (LM) images showing viable evaginated (E) PCs, invaginated (I) unstained PCs, and red dead (D) PCs after 5 min staining with 0.1% eosin stain. H, hooks; C, calcareous corpuscles. **(A and B)** ×100, **(C)** × 200, and **(D)** ×400.

The collected PCs were tested for viability by the eosin exclusion test. Data showed that 97 ± 0.9% of PCs were still viable, and more than 80% exhibited distinct muscular movements. However, the majority was invaginated, and 10% to 20% were evaginated. In addition, the rostellum and suckers were visible (Fig 4).

### 3.4. *In vitro* protoscolicidal activities of Eug and Eug-NE

Control PCs cultured in PBS containing 0.1% DMSO showed minimal differences in PC viability until the 9th day of incubation, followed by gradual total death of all PCs observed on the 17th day of the experiment.

**Fig 5:**
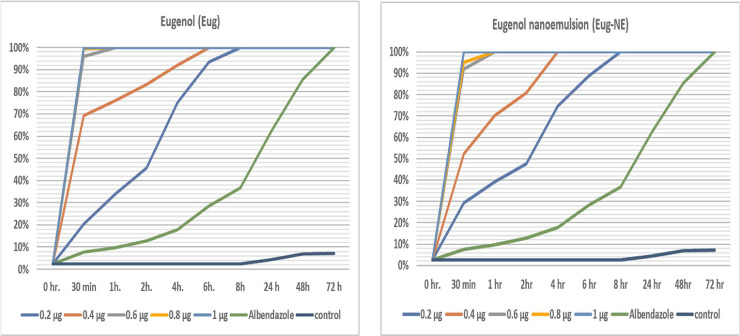
Mortality rate of PCs with various concentrations of Eug and Eug-NE at different exposure periods.

**Fig 5** represents the mortality rates and scolicidal effects of different concentrations of Eug oil and Eug-NE (0.2, 0.4, 0.6, 0.8, and 1 μg/ml) against *E*. *granulosus* PCs at different exposure times (30 min and 1 h, 2, 4, 6, 8, 24, 48, and 72 h). Eug and Eug-NE exhibited significant scolicidal activities compared to controls at all concentrations (*P* < 0.05). These results indicated that Eug and Eug-NE nearly showed similar scolicidal effects at all concentrations, followed by ABZ. Eug oil and Eug-NE at 1 μl/ml showed a profound activity against PC, killing 100% of PCs after 30 min of the experiment, whereas ABZ (800 μg/ml) exhibited a delayed scolicidal activity, killing 100% of PCs after 72 h. The least tested concentration of Eug and Eug-NE was 0.2 μl/ml, which induced a 100% fatality rate after 8 h exposure (S1 Table).

Remarkably, the proven efficacy of Eug and Eug-NE was time-dependent. The mortality rate was significantly higher with increasing exposure times than the concentration. Regarding Eug-NE, there was a strong positive linear correlation between the activity and exposure time (*r* = 0.701) and a moderate positive correlation with the concentration (*r =* 0.554). In contrast, the scolicidal activity of Eug showed a moderate positive correlation with exposure time (*r =* 0.428) and a weak positive correlation with concentration (*r* = 0.341). This was statistically significant (*P* < 0.05; Table 1).

**Table 1:** Correlation between the concentration and exposure time of the scolicidal efficacy of Eug-NE and Eug against hydatid PCs.

	Eug-NE		Eug	
	*r* *	*P* **	*r* *	*P* **
**Efficacy and exposure time**	0.701	0.012	0.428	**0.009**
**Efficacy and concentration**	**0.554**	**0.014**	**0.341**	**0.011**

*Spearman’s rank correlation coefficient.

**Based on normal approximation.

**Fig 6:**
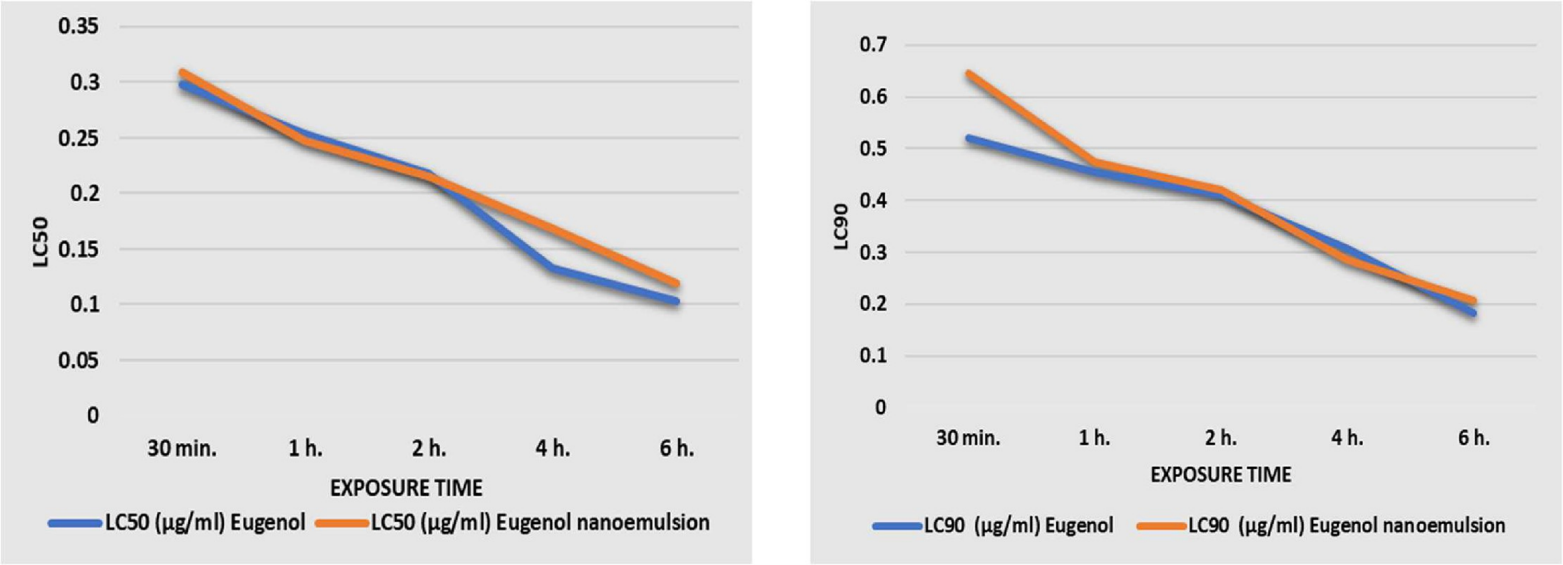
LC_50_ and LC_90_ of Eug and Eug-NE at different exposure periods.

In addition, LC_50_ and LC_90_ of Eug and Eug-NE were calculated at different exposure times. There was no significant difference between LC_50_ and LC_90_ of Eug and Eug-NE at different times, except that, after 6 h, LC_90_ showed a significant difference between the two formulations (Fig 6). These results indicated that LC_50_ and LC_90_ decreased with prolongation of time, but mortality rates increased.

### 3.5. Structural and ultrastructural changes in hydatid cyst PCs induced by Eug and Eug-NE

**Fig 7:**
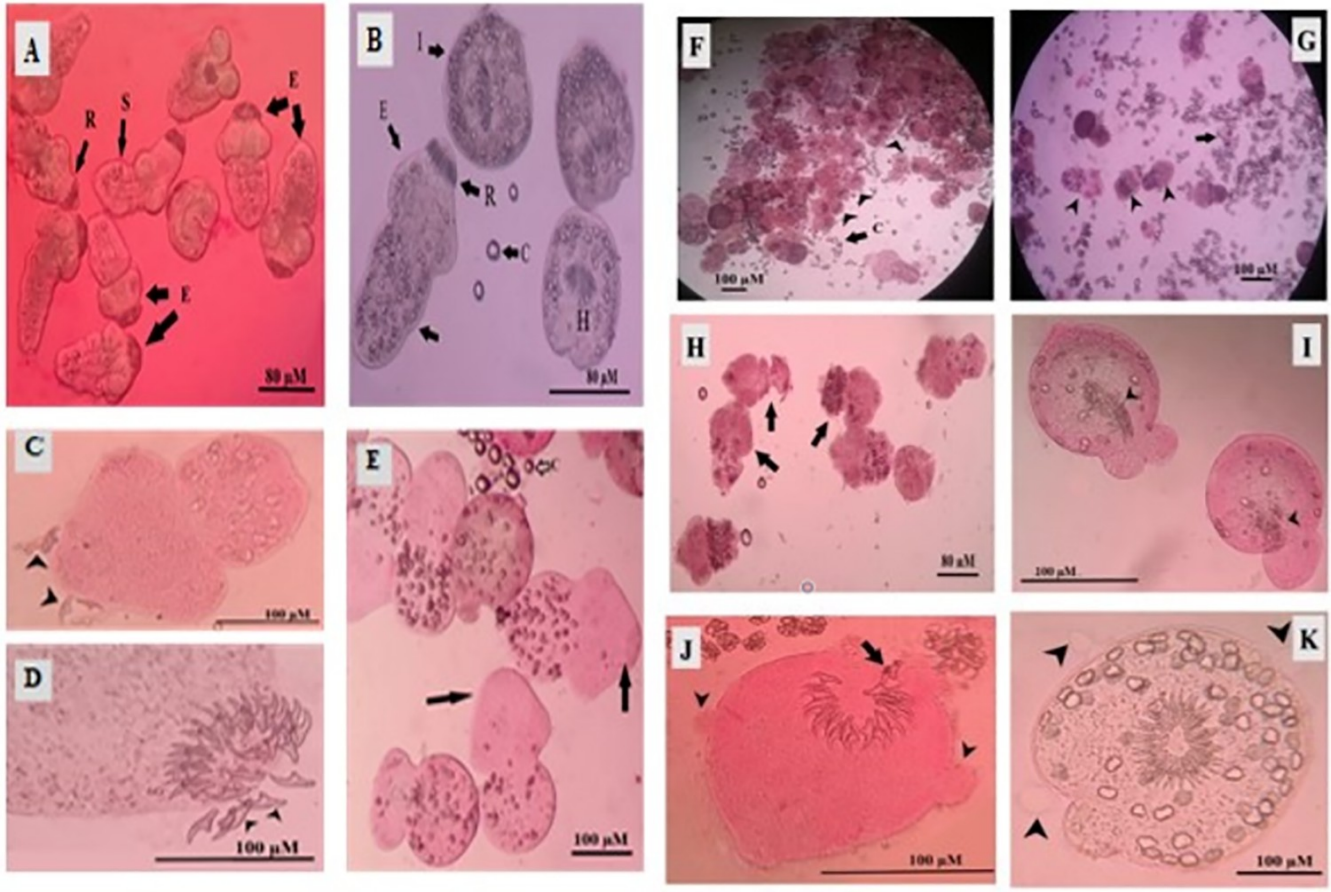
LM changes of PCs stained with 0.1% aqueous eosin stain after 24 h incubation with 0.2 μl/ml Eug, Eug-NE and, ABZ. **(A and B)** Control PCs. **(A)** ×10 and **(B)** ×200. Red PCs are dead. **(C–E)** Dead PCs after incubation with Eug-NE. Note the disorganization of rostellar hooks (arrowheads). **(C)** ×400 and **(D)** ×1000. Distortion of the soma region with a complete loss of hooks (arrows). **(E)** ×200. **(F–I)** Dead PCs after incubation with Eug. **(F)** Note aggregation and adherence of PCs (arrowheads; ×60). **(G)** Distortion of protoscolicidal morphology (arrowheads) with leakage of their content (arrow; ×100). **(H)** Distortion of the soma region with a complete loss of hooks (arrows; ×100). **(I)** Disorganization of hooks (arrowheads; ×400). (**J and K)** Altered PCs after incubation with ABZ. **(J)** Dead protoscolex with several blebs in the tegument (arrowheads) and disorganization of hooks (arrow; ×400). **(K)** Viable protoscolex with several blebs in the tegument (arrowheads; ×400).

**Fig 8:**
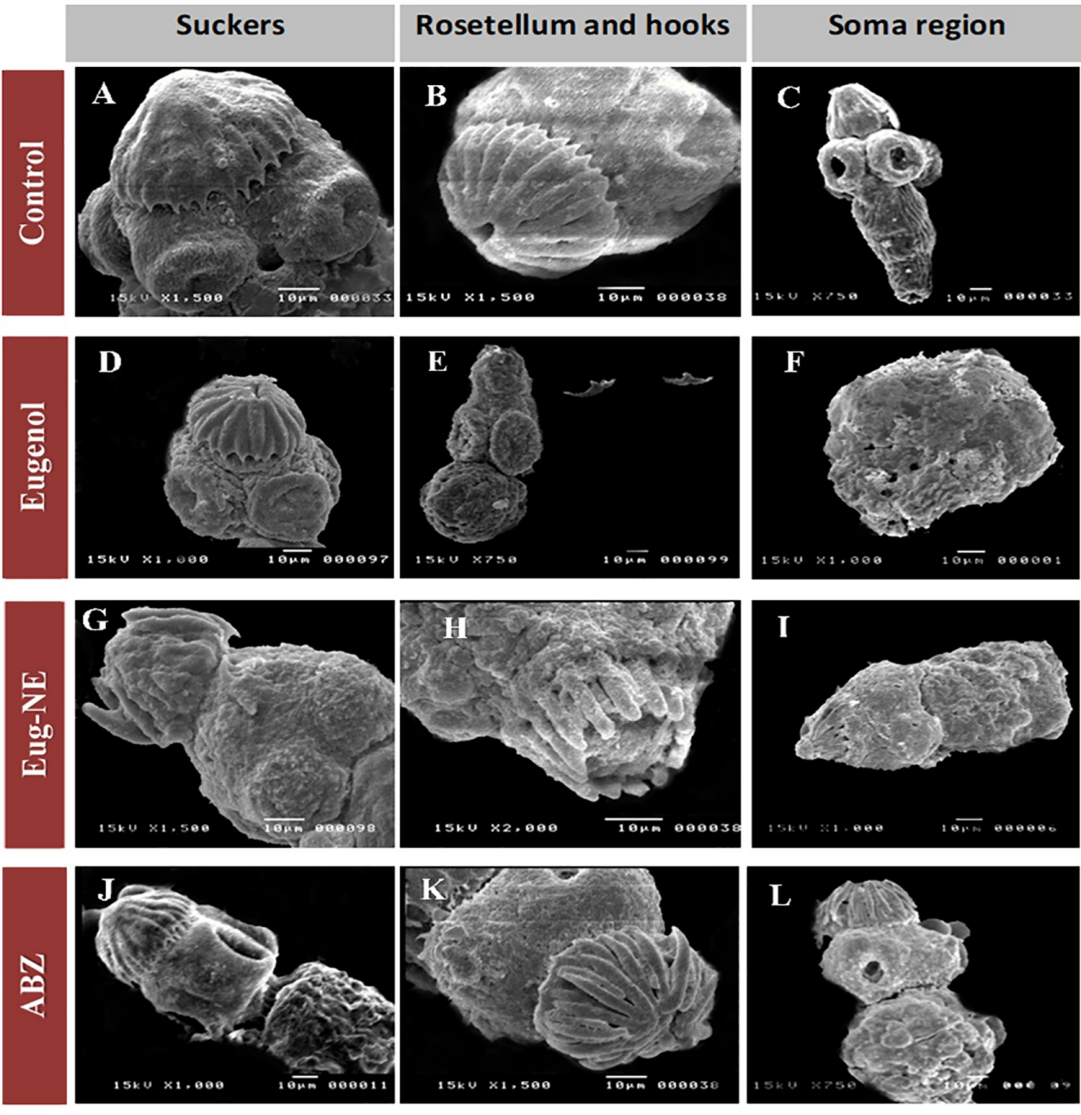
SEM images of *E*. *granulosus* control and treated PCs after 24 h incubation with Eug, Eug-NE, and ABZ. **(A–C)** Evaginated control PCs. **(A** and **B)** ×1500 and **(C)** ×750. **(D–F)** Altered PCs after 24 h incubation with 0.2 μl/ml Eug. A complete loss of rostellar hooks and disruption of suckers can be observed. Contraction of the soma region with the shedding of microtriches and loss of tegumental integrity in the form of holes. **(E)** ×750 and (**D** and **F**) ×1000. **(G–I)** PCs treated with 0.2 μl/ml Eug-NE. The tegument is markedly damaged with loss of microtriches. Disruption of suckers and disorganization of hooks with partial or complete loss of them can be observed. **(G)** ×1500, **(H)** ×2000, and **(I)** ×1000). **(J–L)** Treated PCs with 800 μg/ml ABZ showed disorganization of rosetellar hooks and minimal disruption of suckers with tegumental changes as blebs could be observed. **(J)** ×1000, **(K)** ×1500, and **(L)** ×750.

Inspection of control PCs by LM and SEM revealed that they are small round to oval structures in invaginated PCs covered by intact tegument, and the origin of rostellum appeared as a halo on their anterior part. Evaginated PCs showed well-organized hooks on the rostellar region with rounded suckers, neck and body regions with intact tegument (Figs 7A, 7B and 8A–8C). After 24 h incubation, control PCs remained viable without obvious morphological changes by either LM or SEM. However, upon treatment with Eug, Eug-NE (0.2 μl/ml), and ABZ (800 μl/ml) for 24 h, microscopic morphological changes became more evident. Dead PCs with distorted shape, disorganization of rostellar hooks, and a complete loss of hooks were observed with the addition of Eug and Eug-NE. Also, aggregation and adherence of dead PCs and distortion of the soma region were detected. PCs incubated with ABZ at the same period showed several blebs in the tegument with disorganization of hooks (Fig 7C–7K).

Similar changes were also observed by SEM after 24 h incubation with the treated drugs. PCs incubated with 0.2 μl/ml Eug showed a contraction of the soma region and extensive tegumental damage with loss of integrity in the form of holes. Disorganization of rostellar hooks and a complete loss of hooks with disruption of suckers can also be observed (Fig 8D–8F). After incubation of PCs with 0.2 μl/ml Eug-NE, marked tegumental changes were observed with the formation of blebs and loss of its integrity. Disruption of suckers and the disorganization of hooks with partial or complete loss were other findings (Fig 8G–8I). Treatment of PCs with ABZ induced tegumental changes, stromal blebs with disorganization of rosetellar hooks, and disruption of suckers (Fig 8J–8L).

## 4. Discussion

CE remains a significant zoonotic disease worldwide, affecting mainly the liver of the host. Surgical removal of cysts is recognized as the first choice of management in complicated cases. However, local recurrence and secondary dissemination are common complications [53]. Therefore, inactivating PCs by scolicidal agents was introduced as an alternative to surgical approaches and was associated with high efficacy with minimum side effects [54]. Moreover, chemotherapy could be applied for inoperable patients or as a complementary treatment or adjuvant to surgery (preoperative, postoperative, or both) [55].

The main criteria for an effective scolicidal agent are to achieve higher efficacy at lower concentrations in a short exposure time, long-term stability in the hydatid fluid, lower toxicity, and easy preparation [56]. Up to date, ABZ is the standard drug used for CE treatment. However, its unsatisfactory response, serious side effects, and development of protoscolicidal resistance limited its use [13,14,16]. Furthermore, several scolicidal agents widely used in CE management also showed undesirable side effects, limiting their application. Many adverse side effects were reported for 20% hypertonic saline, 0.5% silver nitrate, 0.5% to 1% cetrimide, and ethyl alcohol [13]. Hence, developing alternative scolicidal agents from natural essential oils and nano-preparations with limited side effects and high potency would be a valuable achievement [57].

This study showed the high effectiveness of Eug-NE and Eug oil against hydatid PCs compared to ABZ with a highly significant difference (*P* < 0.05). Both Eug and Eug-NE nearly have similar protoscolicidal effects at all used concentrations. Their highest scolicidal activities (100% mortality rate) were recorded at 1 μl/ml after 30 min incubation, whereas that of ABZ (800 μg/ml) was after 72 h. Both Eug and Eug-NE showed time- and dose-dependent effects.

These results were consistent with previous reports showing the high efficacy of Eug as a strong anthelmintic against different trematodes and nematodes, such as the adult fluke *F*. *gigantica* and the gastrointestinal nematode *H*. *contortus* [25]. Also, Eug exhibited potent antiprotozoal effects on *G*. *lamblia*, *P*. *falciparum*, and other protozoal infections [27–30,32,58].

As per the results, there was no significant difference between eugenol and its nanoemulsion formulation and both exhibited similar anthelmintic and scolicidal activities in the current study. This may be attributed to the presence of aggregates of oil nano-droplets in the formulation as evidenced by the large PDI value of the formulated Eug-NE. Moreover, these aggregates reduce the surface area of the oil exposed to the parasite larvae and hence, reduce the overall activity.

In this study, Eug essential oil was investigated for the first time against the cestode *E*. *granulosus* PCs, showing a potent scolicidal effect (100%) at very low concentrations and after a short period (1 μl/ml after 30 min). Remarkably, Eug proved a higher efficacy compared to several essential oils previously investigated against hydatid cyst PCs, such as *Rosmarinus officinalis* with an effective dose of 1 mg/ml after 8 days of incubation, *Thymus vulgaris* (1 mg/ml) after 7 days [41], *Trachyspermum ammi* [59], *Nigella sativa* [60], and *Satureja khuzistanica* essential oils [61] (10 mg/ml) after 10 min, and *Myrtus communis* (100 μl/ml) [62], *Pistacia vera* [8], and *Curcuma longa* essential oils (200 μl/ml) [63] after 5 min, and 0.5% of *Eucalyptus globulus* essential oil after 3 min [64]. In addition, Eug proved high scolicidal efficacy in very small doses than that was observed with *Zataria multiflora* essential oil emulsion against hydatid PC [65] and the germinative cells [66] (20 μl/ml for 10 min and 8 mg/ml for 7 min, respectively).

Interestingly, Eug is the principal component of clove (*S*. *aromaticum***)**. This study addressed the higher potency of Eug as a scolicidal agent than did *S*. *aromaticum* assayed by Selles et al. (2020), who reported that *S*. *aromaticum* essential oil induced a 100% mortality rate at the dose of 15 μl/ml [5]. This could be attributed to the presence of the minor components of *S*. *aromaticum* essential oil affecting the activity of Eug. Marked differences between the efficacy of different essential oils and their main components were also reported in previous studies. Fabbri et al. (2018) reported that beta-myrcene is more effective than *R*. *officinalis* essential oil (rosemary)[67]. Also, Pensel et al. (2014) reported a high protoscolicidal effect of thymol than *T*. *vulgaris* and *Origanum vulgare* essential oils [68].

Several studies have used nano preparations and essential oil nanoemulsions as novel and alternative approaches showing remarkable protoscolicidal effects like that achieved by Moazeni et al. and others who studied the antihydatid activity of nanoemulsions of *Zataria multiflora* [65,69] and *Satureja hortensis* [70]. However, metal nanoparticles (NPs), such as those of silica, copper, iron, and zinc [71], gold NPs [72], biosynthesized silver NPs [73], zinc oxide NPs [55], and biogenic selenium NPs showed lesser scolicidal effects than Eug-NE [57].

Eug and Eug-NE produced evident morphological changes as observed by LM and SEM. The disintegration of PCs accompanied by disruption of suckers and a complete loss of hooks were observed by SEM. The mechanism of action of Eug could be argued to its ability to disintegrate the cell membrane by deformation of membrane macromolecules, causing protein denaturation and modification of cell membrane permeability [18]. Pasqua et al. (2007) reported that Eug modified the cell membrane’s fatty acid profile, resulting in a large increase in concentrations of low molecular weight fatty acids producing disruption of cellular permeability [74]. Also, Hyldgaard et al. (2012) showed that Eug inhibits cellular enzymes, including protease, histidine carboxylase, amylase, and ATPase [75].

The exact mechanism of action of Eug-NE as a protoscolicidal agent has not been verified. Generally, the nanoemulsion delivery system for Eug essential oil could improve the bioavailability and the efficacy of its active compounds through their solubilization into nanodroplets [76]. Therefore, it possibly enhances their interaction with the Protoscolix cell membranes by different routes: i) increasing the surface area and passive transport across the cell membrane [77]; ii) increasing the concentration of active essential oil at the targeted site of action through the fusion of nanodroplets with the phospholipid layer of the cell membrane [78]; iii) the thermodynamic stability of nanoemulsion provides a sustained release of essential oil over time, thus enhances its activity [79]. Our results agreed with other studies that documented the efficacy of Eug-NE as a potential leishmanicidal agent with immunostimulatory functions without adverse side effects [80]. Also. Eug-NE showed an antibacterial activity against *Staphylococcus aureus* and *E*. *coli* compared to Eug alone. Ghosh et al. (2014) demonstrated that Eug-NE exhibited bactericidal activity against *S*. *aureus*, and the use of fluorescence microscopy confirmed the alteration in membrane permeability with subsequent bacterial cell death [81]. In contrast, Hu et al. (2016) reported that Eug and Eug-NE have similar bactericidal activities against *L*. *monocytogenes* and *S*. *enteritidis* [38].

Therefore, given the merits of safety and high effectiveness against hydatid protoscoleces exceeding the traditional scolicides, novel nanoemulsion preparations could be introduced for the potential use in clinical settings or adjuvant to surgery.

## 5. Conclusions

This study demonstrated the significant scolicidal activities of Eug essential oil and Eug-NE formulation against hydatid cyst PCs compared to the control drug, ABZ. Both proved their efficacy in very small doses at short periods of treatment with the least cytotoxicity level. The study suggested the potential value of Eug and Eug-NE as promising scolicidal agents. However, further studies, including *in vivo* assays and more mechanistic studies are necessary to fully determine the potential of these compounds for the prevention and treatment of cystic echinococcosis. Also, additional work is required to explore the physical stability of the formulated nanoemulsion in the presence of another cosurfactant, such as lecithin, and its effects on hydatid cysts.

## Supporting information

S1 Table Scolicidal effects of various concentrations of Eug-NE and Eug on PCs at different exposure times. *Values of the positive control were significant with negative control (*P* < 0.05). **Eug-NE/Eug values were significant with the positive control (PC) (ABZ; *P* < 0.05). Eug-NE/Eug values versus the negative control (NC) were significant (*P* < 0.05). ^a^One-way ANOVA. ^b^Repeated-measures ANOVA.
(PDF)
