# Supplementary material for: Evaluation of the scolicidal activities of eugenol essential oil and its nanoemulsion against protoscoleces of hydatid cysts
Source: PLoS One. 2021 Nov 11;16(11):e0259290. doi: 10.1371/journal.pone.0259290 (PMC8584737; doi:10.1371/journal.pone.0259290)
Supplement: S1 Table — *Values of the positive control were significant with negative control (P < 0.05). **Eug-NE/Eug values were significant with the positive control (PC) (ABZ; P < 0.05). Eug-NE/Eug values versus the negative control (NC) were significant (P < 0.05). aOne-way ANOVA. bRepeated-measures ANOVA. (PDF) [file pone.0259290.s001.pdf]

S1 Table: Scolicidal effects of various concentrations of Eug-NE and Eug on PCs at different exposure times.

IP exposure time	Mortality rate% (scolicidal efficacy; mean ± SD)											
	ABZ (PC)	DMSO (NC)	Eug-NE					Eug					*P*. value
			0.2 µl/ml	0.4 µl/ml	0.6 µl/ml	0.8 µl/ml	1 µl/ml	0.2 µl/ml	0.4 µl/ml	0.6 µl/ml	0.8 µl/ml	1 µl/ml	*P*[a]
**30 min**	7.7 ± 0.5	2.5 ± 0.2	29.2 ± 1.6	52.3 ± 1.8**	92 ± 2.3**	95 ± 3.1**	100 ± 0**	20.5 ± 1.6	69.4 ± 2.4**	96 ± 2.2**	99.3 ± 1.1**	100 ± 0**	<0.001
**1 h**	9.7 ± 0.86	2.5 ± 0.2	39.3 ± 1.8	70.2 ± 1.8**	100 ± 0**	100 ± 0**	100 ± 0**	33.8 ± 1.6	75.9 ± 2.0**	100 ± 0**	100 ± 0**	100 ± 0**	<0.001
**2 h**	12.9 ± 0.7	2.5 ± 0.2	47.5 ± 1.7**	81.0 ± 1.8**	100 ± 0**	100 ± 0**	100 ± 0**	45.7 ± 1.7**	83.4 ± 1.9**	100 ± 0**	100 ± 0**	100 ± 0**	<0.001
**4 h**	17.9 ± 1.7	2.5 ± 0.2	74.6 ± 1.9**	100 ± 0**	100 ± 0**	100 ± 0**	100 ± 0**	75.3 ± 1.8**	92.1 ± 2.8**	100 ± 0**	100 ± 0**	100 ± 0**	<0.001
**6 h**	28.5 ± 1.0	2.5 ± 0.2	88.9 ± 2.6**	100 ± 0**	100 ± 0**	100 ± 0**	100 ± 0**	93.5 ± 2.3**	100 ± 0**	100 ± 0**	100 ± 0**	100 ± 0**	<0.001
**8 h**	36.8 ± 1.6	2.5 ± 0.2	100 ± 0**	100 ± 0**	100 ± 0**	100 ± 0**	100 ± 0**	100 ± 0**	100 ± 0**	100 ± 0**	100 ± 0**	100 ± 0**	<0.001
**24 h**	62.6 ± 2.2*	4.4 ± 0.4	100 ± 0**	100 ± 0**	100 ± 0**	100 ± 0**	100 ± 0**	100 ± 0**	100 ± 0**	100 ± 0**	100 ± 0**	100 ± 0**	<0.001
**48 h**	85.5 ± 1.9*	6.8 ± 0.2	100 ± 0	100 ± 0	100 ± 0	100 ± 0	100 ± 0	100 ± 0	100 ± 0	100 ± 0	100 ± 0	100 ± 0	<0.001
**72 h**	100 ± 0.0*	7.3 ± 0.4	100 ± 0	100 ± 0	100 ± 0	100 ± 0	100 ± 0	100 ± 0	100 ± 0	100 ± 0	100 ± 0	100 ± 0	<0.001
P value[b]	<0.001	0.789	0.008	0.028	0.782	0.895	1.000	0.001	0.625	0.908	0.927	1.000	<0.001
